# Cellular and Molecular Networking Within the Ecosystem of Cancer Cell Communication via Tunneling Nanotubes

**DOI:** 10.3389/fcell.2018.00095

**Published:** 2018-10-02

**Authors:** Emil Lou, Edward Zhai, Akshat Sarkari, Snider Desir, Phillip Wong, Yoshie Iizuka, Jianbo Yang, Subbaya Subramanian, James McCarthy, Martina Bazzaro, Clifford J. Steer

**Affiliations:** ^1^Division of Hematology, Oncology and Transplantation, Department of Medicine, University of Minnesota, Minneapolis, MN, United States; ^2^Department of Integrative Biology and Physiology, University of Minnesota, Minneapolis, MN, United States; ^3^Division of Gastroenterology, Hepatology and Nutrition, Department of Medicine, University of Minnesota, Minneapolis, MN, United States; ^4^Division of Gynecologic Oncology and Women's Health, Department of Obstetrics and Gynecology, Masonic Cancer Center, University of Minnesota, Minneapolis, MN, United States; ^5^Department of Laboratory Medicine and Pathology, University of Minnesota, Minneapolis, MN, United States; ^6^Department of Surgery, University of Minnesota, Minneapolis, MN, United States

**Keywords:** angiogenesis, cancer ecosystems, cancer pathophysiology, intercellular communication, intercellular transfer, tumor microenvironment, tumor microtubes, tunneling nanotubes

## Abstract

Intercellular communication is vital to the ecosystem of cancer cell organization and invasion. Identification of key cellular cargo and their varied modes of transport are important considerations in understanding the basic mechanisms of cancer cell growth. Gap junctions, exosomes, and apoptotic bodies play key roles as physical modalities in mediating intercellular transport. Tunneling nanotubes (TNTs)—narrow actin-based cytoplasmic extensions—are unique structures that facilitate direct, long distance cell-to-cell transport of cargo, including microRNAs, mitochondria, and a variety of other sub cellular components. The transport of cargo via TNTs occurs between malignant and stromal cells and can lead to changes in gene regulation that propagate the cancer phenotype. More notably, the transfer of these varied molecules almost invariably plays a critical role in the communication between cancer cells themselves in an effort to resist death by chemotherapy and promote the growth and metastases of the primary oncogenic cell. The more traditional definition of “Systems Biology” is the computational and mathematical modeling of complex biological systems. The concept, however, is now used more widely in biology for a variety of contexts, including interdisciplinary fields of study that focus on complex interactions within biological systems and how these interactions give rise to the function and behavior of such systems. In fact, it is imperative to understand and reconstruct components in their native context rather than examining them separately. The long-term objective of evaluating cancer ecosystems in their proper context is to better diagnose, classify, and more accurately predict the outcome of cancer treatment. Communication is essential for the advancement and evolution of the tumor ecosystem. This interplay results in cancer progression. As key mediators of intercellular communication within the tumor ecosystem, TNTs are the central topic of this article.

## Introduction

Malignant tumors are heterogeneous and highly dynamic ecosystems. Cellular communication is a critical component of heterotypic and homotypic interactions in the complex, ever-changing tumor microenvironment. Tunneling nanotubes (TNTs) are long filamentous actin-based cellular protrusions that contribute to these interactions, with a special role in long-range communication (Rustom et al., 2004; Onfelt et al., 2005; Eugenin et al., 2009; Gousset et al., 2009, 2013; Chauveau et al., 2010; Plotnikov et al., 2010; Lou et al., 2012; Pasquier et al., 2013; Zhang and Zhang, 2015). While substantial progress has been made in understanding the function of TNTs over the past 5 years, there remain many unknowns, such as whether or not there exist a single or multiple sets of structural or other biomarkers that are characteristic of and specific to TNTs across cell types. In addition, many of their behavioral characteristics remain unclear.

By using a systems biology approach to characterize TNTs, we can further shed light on the interactions that are mediated within the tumor microenvironment. These interactions include not only cell-to-cell communication among malignant cells, but also interactions between malignant and stromal cells within the extracellular matrix, including vascular endothelial cells and cancer-associated fibroblasts. These are just two examples of stromatous cell types that are susceptible to potential reprogramming. Downstream effects of cellular reprogramming that result from indirect or direct cell communication have strong implications in altering not only the growth of tumor but also its metastatic potential.

Here, we discuss our theory that investigating the tumor ecosystem by focusing on long-range communication via TNTs will yield novel perspectives on their role in the evolution of cancer. There is strong interest in the identification of stimulatory factors and molecular machinery of TNT formation and maintenance as potential biomarkers of the disease. We follow up on our previous report that hypoxia—a physiologic condition that is characteristic of the tumor microenvironment and one that is heavily associated with metabolic dysfunction and tumor invasiveness—induces TNTs in ovarian cancer cells (Desir et al., 2016), by confirming this in another model system (colon cancer). Taking this a step further, we hypothesize that the conditions of hypoxia are not only favorable for the formation of TNTs but also that these TNTs in turn are capable of propagating hypoxia-inducible factor-1α (HIF-1α) and vascular endothelial growth factor (VEGF) between connected cells to stimulate angiogenesis. Another form of heterotypic interaction occurs between malignant cells and the hematologic system, including red blood cells (RBCs) and platelets, which themselves have been found to form pseudopodia-like protrusions that may in fact signify TNTs. We also examine other potential candidate components of the plasma membrane (proteoglycan chondroitin sulfate proteoglycan 4, or CSPG4) and cellular machinery of motility (UNC-45A) in relation to cancer cell TNTs. Another form of transmembrane protein, the nucleoside transporter human equilibrative nucleoside transporter 1 (hENT1), whose expression is associated with more efficient cell-to-cell diffusion of fluoropyrimidine chemotherapeutic drugs, is also of interest; we examine whether this protein varies with TNT formation. The growing body of evidence that TNTs play a role in connecting cells within the cancer ecosystem provides a basis for expanding potential applications of TNTs to explain fundamental processes in cancer as well as in normal cell function. The clinical relevance of this field is focused on TNTs as structural components of cells that are potential targets for drug therapy or for other targeting strategies.

## TNT formation is upregulated by hypoxic conditions in multiple forms of cancer, including colon cancer

**Figure 1:**
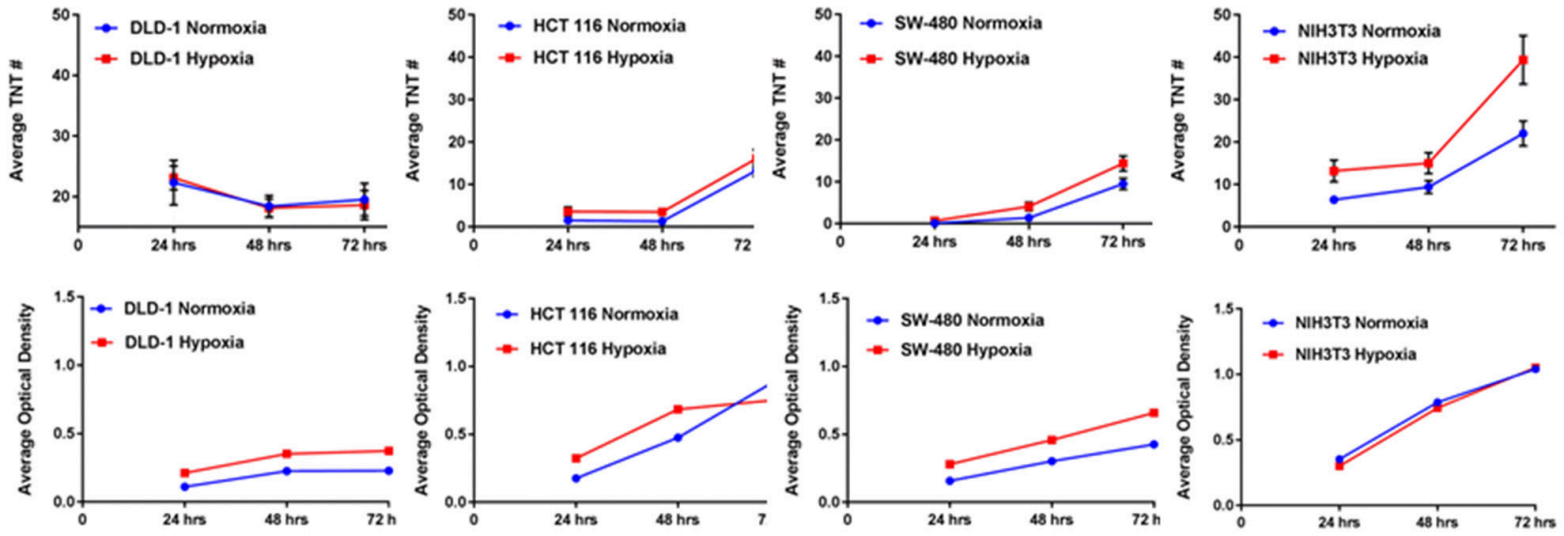
Hypoxia induces TNT formation in colon cancer cells, independent of alterations in cell proliferation. Graphs demonstrate changes in average number of TNTs at 24 h, 48 h, and 72 h (top row) under normoxic vs. hypoxic conditions and average optical density as a surrogate for cell proliferation at the same time points (bottom row) for the colon cancer cell lines DLD-1, HCT-116, and SW-480 and for the comparison of the fibroblast cell line NIH 3T3. Materials and Methods section for experiments shown in the figure is available in the Supplementary Material.

Tunneling nanotubes are a form of cellular stress response to conditions that favor physiologic and metabolic dysregulation (Lou et al., 2012; Wang and Gerdes, 2012). In 2016, we reported that hypoxia, a state that is characteristic of invasive malignancies and the solid tumor microenvironment, induced a higher rate of TNT formation in ovarian cancer cells (Desir et al., 2016). Moreover, the effect was most pronounced in chemoresistant ovarian cancer cells even after treatment with a compound that effectively suppressed TNTs in sensitive cells. To provide further confirmation that this observation could be applied to other cancer cell types, we compared TNT formation under conditions identical to colon cancer cells subjected to hypoxia vs. normoxia. We used three different cell lines (SW480, HCT-116, and DLD-1), in addition to two non malignant cell types, the colon adenoma (premalignant)-derived cell line AAC1 and fibroblasts (NIH 3T3 cell line). Following the same experimental approach that previously showed upregulated HIF-1α expression (Desir et al., 2016), we detected TNT formation when all three of the colon cancer cell lines were cultured in hypoxic conditions (Figure 1). The AAC1 cells failed to form TNTs under normoxic conditions, and this lack of TNT formation was again observed even under hypoxic conditions. We also confirmed that NIH 3T3 fibroblasts readily formed TNTs under both conditions. Upon analysis of absolute numbers of TNTs it was found that SW480 and HCT-116 carcinoma cells and NIH 3T3 cells formed more TNTs under hypoxic than normoxic conditions, but DLD-1 cells demonstrated no difference. To account for alterations in cell metabolism and cell viability, we again used absorbance as a surrogate for the direct quantification of cells using the Cell Counting Kit (see Methods section for details); this procedure ensured that differences in numbers of TNTs could not be attributed to changes in cell proliferation under differing conditions. After accounting for altered cell metabolism from hypoxia, the differences in TNT formation were most profound for the HCT-116 and NIH 3T3 cells by 72 h. Conversely, TNT formation was negligible for SW480 cells. In contrast, DLD-1 cells were less responsive to hypoxia; overall, the TNT/absorbance ratio was actually lower for these cells after hypoxic exposure at all three time points (24, 48, and 72 h).

## Heterotypic interactions between malignant cells and stroma mediated by TNTs: deciphering communication between vascular, hematologic, and immune cells

**Figure 2:**
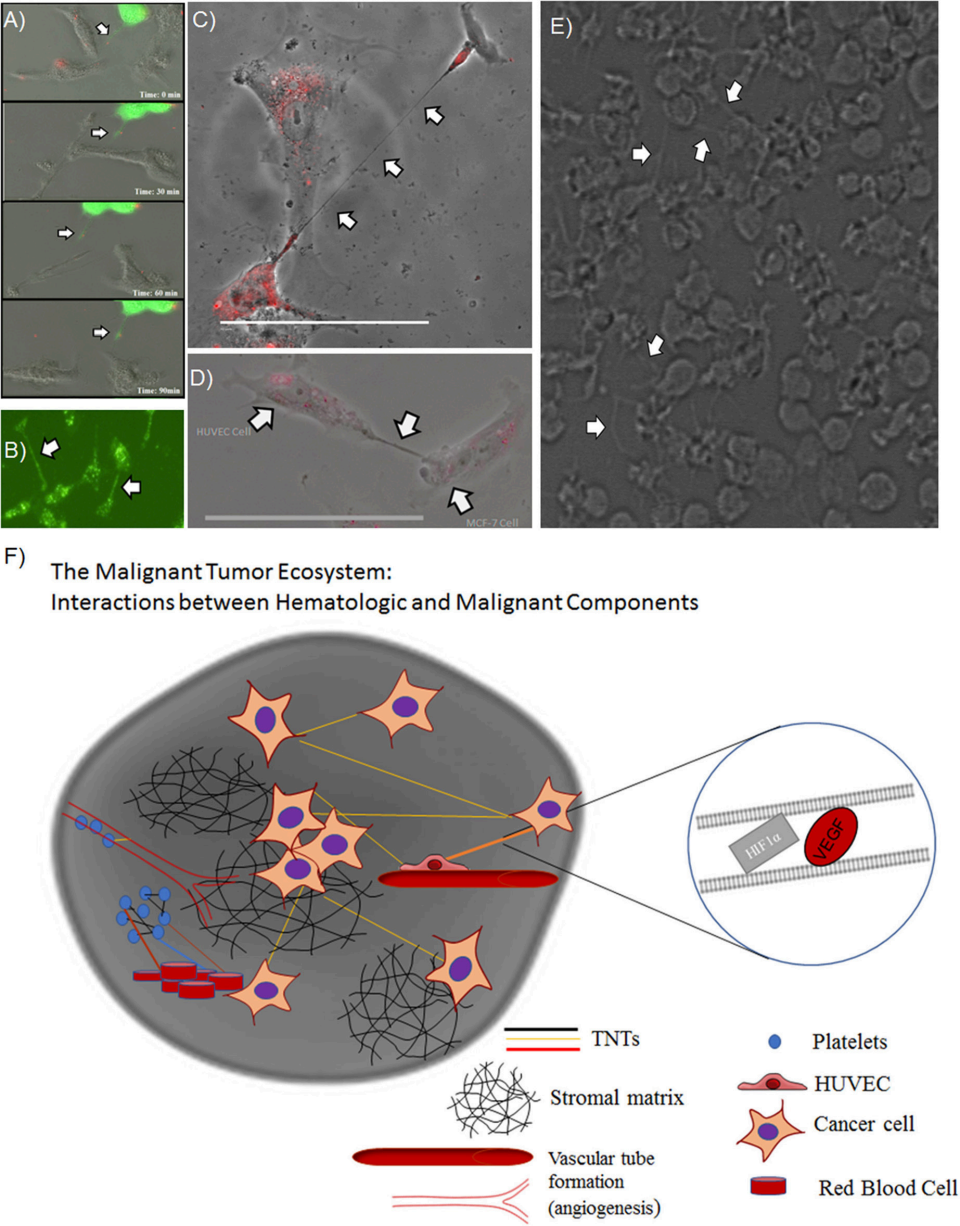
TNTs induced by hypoxia stimulate a potential positive feedback loop by mediating intercellular transfer of HIF-1α and VEGF. Heterotypic forms of TNTs include malignant cell-endothelial cell TNT formation, as well as potential TNT formation among clusters of platelets. **(A)** Composite of images from a time-lapse series demonstrating intercellular transfer of GFP-tagged HIF-1α via a TNT that connects SKOV3 ovarian cancer cells. **(B)** SKOV3 cells expressing GFP-VEGF form TNTs that transfer VEGF. **(C)** HUVECs stained with DiI connected via a long TNT. **(D)** Heterotypic TNT formation between a HUVEC (left) and a breast cancer cell (MCF-7, on the right). **(E)** A cluster of platelets cultured *in vitro* forming many fine pseudopodia-like protrusions representing potential TNTs. **(F)** Schematic demonstrating potential interplay among microthrombi formed by platelets and/or RBCs communicating via TNTs, in the same ecosystem as malignant cells communicating with TNTs. Scale bars = 100 μm. Materials and Methods section for experiments shown in the figure is available in the Supplementary Material.

Overall, these results provided confirmation and support for our prior work demonstrating that hypoxic conditions induce metabolic stress that results in upregulated TNT formation in some invasive cancer cells but not in others. Nonetheless, the confirmation of the hypoxic effects of TNTs on both malignant and stromal (fibroblast) cells provides further support for an important spatial and developmental timing niche for TNTs in the process of tumor growth. This prospect opens the door to deciphering the potential role of these TNTs in mediating heterotypic matrix-cancer cellular interactions in invasive, hypoxic tumors. A prime translational example of this context is angiogenesis, the process of vascular tube formation that arises in reaction to hypoxia that stems from diminished diffusion of oxygen that usually permeates toward the central portions of tumors. Angiogenesis is central to vascular interactions within the cancer ecosystem. As tumors grow and oxygen levels decrease, malignant cells secrete soluble VEGF that diffuses across the intercellular space and is taken up by the vascular endothelium via its corresponding receptor. This process results in angiogenesis, in which malignant cells essentially direct the construction of their own stromal matrix and mediate a new form of enriched oxygenation through increased blood flow. To date, the secretion and downstream effects of VEGF have been assumed to occur solely by diffusion through the microenvironment. However, it is also conceivable that TNTs can form between malignant and vascular endothelial cells and act as conduits for VEGF transfer. If true, this concept would expand our view of stromal-cancer cell interactions and the understanding of this stage of cancer pathophysiology. The images and schematic in the accompanying figure (Figure 2) provide a glimpse of this potential, as we have observed and confirmed intercellular connection via TNTs that form between malignant and human vascular endothelial cells (HUVEC); furthermore, it was confirmed via time-lapse imaging that these TNTs transfer VEGF and even HIF-1α. This provides a prime example of how malignant cells contained within the extracellular matrix can not only contact but also potentially reprogram other cells, thereby, leading to invasion and metastasis. As a whole, this collective set of interactions would effectively serve as a mammalian and cancer version of quorum sensing (Schertzer and Whiteley, 2011; Doganer et al., 2016) but with the significant addition of TNTs as a more intimate and direct mode of interaction and exchange of information. While the term “quorum sensing” is usually reserved for bacteria, it should also be used to effectively describe the choreography of complex and dynamic interactions and exchange of “social information” among cancer cells by TNTs and other means.

A natural clinical extension of angiogenesis is the fact that cancer provides not just a pro-inflammatory state but also one that is prothrombotic. The transmembrane receptor tissue factor (TF) is known to bind plasma factors that initiate the cascade of events leading to hypercoagulation, and this process is expedited by TF-positive microparticles released by cancer cells (Geddings and Mackman, 2013). For this reason, the risk of venous thromboembolism (VTE) is significantly increased in the presence of cancer, and the development of VTE can potentially be fatal when not diagnosed and treated with anticoagulation therapy in a timely fashion. Part of the biochemical cascade that results in VTE includes activation of thrombin, a serine protease that converts fibrinogen to fibrin. A recent elegant study demonstrated the ability of thrombin to induce TNTs in endothelial cells (Pedicini et al., 2018), providing further support to the notion that TNTs play a previously uncovered role in this cancer-related process.

In addition to heterotypic TNT connections between hematologic, malignant, and vascular endothelial cells, there is also potential for TNTs to connect cell bodies and factors that comprise thromboemboli, including platelets. There are emerging data to support this concept. Platelet aggregation has a strong association with advanced malignancy; the resulting VTE or microthrombi are not just by-products of this cancer-induced inflammatory state. Paraneoplastic thrombocytosis is a known phenomenon in which inflammatory cytokines, such as interleukin-6 (IL-6), released by malignant cells lead to increased synthesis of thrombopoietin and platelet number, which in turn further stimulate tumor growth (Stone et al., 2012). If platelet-tumor cell interactions are direct, rather than dependent on diffusible soluble factors, this form of communication would be highly effective in the relatively enclosed space of the tumor-hematologic interface within the cancer microenvironment. Studies that employ electron microscopy (EM) to examine platelets have led to visualization of podosome-like structures that are composed of actin nodules (Poulter et al., 2015). Moreover, longer slender actin-based protrusions that connect platelets, containing bead-like bulges that may represent transported cargo, have been identified and labeled as pseudopodia or other types of cell protrusions (Junt et al., 2007; Schwertz et al., 2010; van Rooy and Pretorius, 2016). However, in hindsight, some or all of the above forms of protrusions may in fact have been TNTs. In culturing human platelets *in vitro*, we too have identified similar pseudopodia connecting platelets in a fashion identical to TNTs connecting cancer cells (Figure 2). We speculate that TNTs may form and play a role in communication not just between cells but also between anucleate structures, such as RBCs and platelets (Swanepoel and Pretorius, 2013; Olumuyiwa-Akeredolu and Pretorius, 2015).

Platelets and RBCs can interact, and the membrane structure of both of these hematologic components is dictated by lipid content, which includes the formation and maintenance of lipid rafts. In fact, over a decade ago it was reported that tubular budding of RBCs led to the formation of TNTs and that these TNTs function by permitting vesicular transfer between connected erythrocytes (Iglic et al., 2007). Furthermore, our group has previously reported that cells that form TNTs are enriched in lipid rafts, and this finding includes localization of these raft complexes at the base of TNTs (Thayanithy et al., 2014a); a finding that is consistent with the report of tubular budding as an early precursor of TNT formation. The observations and background findings summarized above open new avenues to a potential role of TNTs in benign hematologic studies as well as in malignancy by providing a direct link between intercellular communication and the pro-inflammatory state that induces a higher degree of tumor aggressiveness.

There is a growing body of evidence that TNTs also mediate cellular interactions between malignant cells and immune cells that infiltrate the tumor microenvironment. Correlation of immune infiltration of tumors with patient prognosis and risk of recurrence following definitive treatment is now better recognized. For example, in patients with stages I-III colon carcinoma, the extent of tumor-infiltrating T cells is inversely correlated with risk of recurrence (Pagès et al., 2018). It is, therefore, conceivable that T cells infiltrating the tumor matrix are communicating with each other, with other immune-type cells, vascular endothelium, hematologic cells, and even with the malignant cells themselves via TNTs to enact an antitumor response (Lachambre et al., 2014; Al Heialy et al., 2015). Another example of immune cell TNT formation has been identified in macrophages (Hanna et al., 2017). Tumor-associated macrophages (TAMs) present in colon tumor stroma are associated with more invasive forms of this disease (Zhang et al., 2013). Intercellular cross talk between malignant colon cells and the M2 phenotype of macrophages induces a faster migration of the malignant cells, which in turn secrete cytokines such as IL-10 that promote further differentiation of the macrophages (Zhang et al., 2013). It is conceivable that this form of cross talk could also be mediated by TNTs, which in this scenario are promoting a more aggressive phenotype within the tumor ecosystem. The above represents just two of the many potential possibilities for immune-cancer interactions that are facilitated by TNTs. Whether TNT-mediated interplay between immune cells and other heterogeneous components of the tumor matrix results in a net increase or decrease in metastatic potential is a possibility that needs to be explored.

## Tunneling nanotubes and the dunbar number: uncovering the extent and limitations of intercellular conversations

There is potential merit in learning from and borrowing concepts of social interactions in society to behavior at the cellular level. This approach is compatible with the cancer systems biology approach of examining cells—which themselves are small components of an ever-changing, dynamic, and heterogenous tumor microenvironment—in their greater context rather than in isolation.

**Figure 3:**
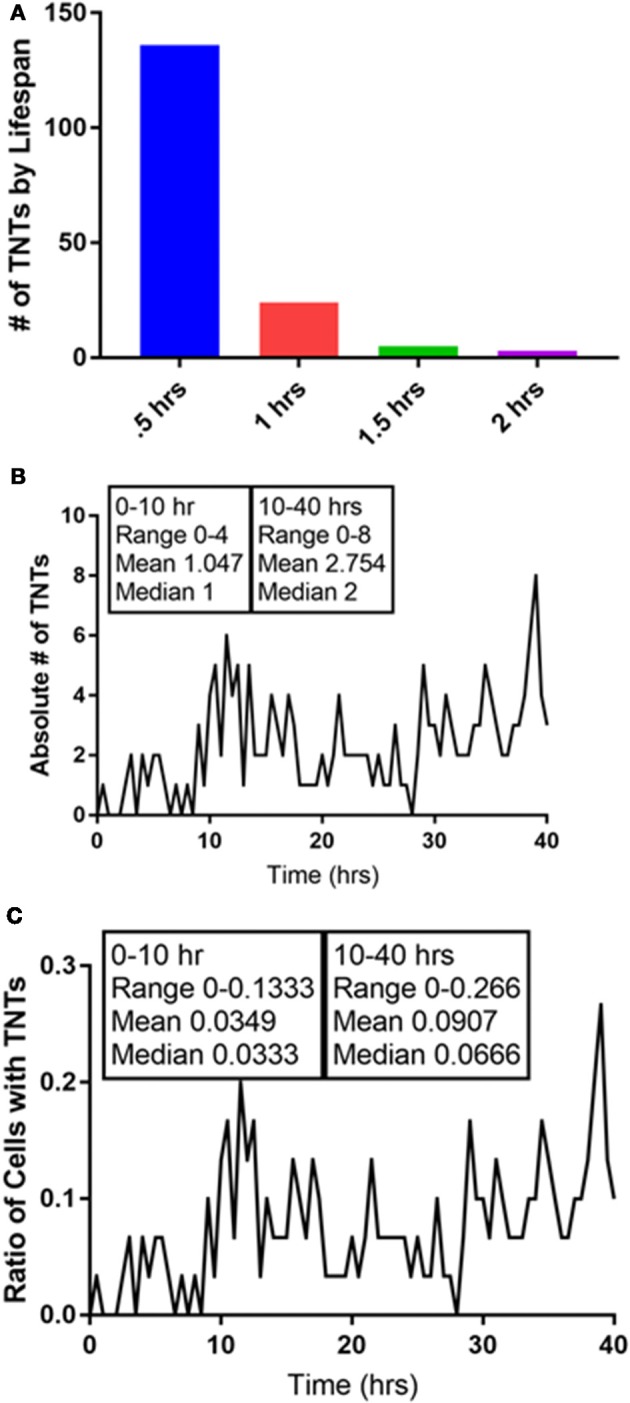
Quantification of the intercellular interactions that occur via TNTs: in search of a Dunbar's number for TNTs. We analyzed a 40-h time-lapse set of images of a coculture of Mg63.2 osteosarcoma cells with hFOB osteoblast cells. **(A)** The duration of TNTs is relatively short, as nearly all of the TNTs that were formed lasted for 1 hour or less. **(B)** The absolute number of TNTs increases after 10 h in culture, as does the ratio of number of cells with TNTs **(C)**. All of these images are presented in video form in Supplementary Video 1. Materials and Methods section for experiments shown in the figure is available in the Supplementary Material.

A unique concept from the field of anthropology and mathematics has emerged in which the number of potential social interactions by a human being is defined and limited. Richard Dunbar, an anthropologist, proposed a very specific and finite number for the potential social interactions by a human: 150 (Dossey, 2017). We found the hypothesis and his rationale to be intriguing and considered that TNTs—the purveyors of connections and communication at the cellular level—might also have a finite number in any given cell system. In our previously reported work, we gained insight into the heterogeneity of intercellular interactions and communication via TNTs that formed between chemoresistant and chemosensitive ovarian cells as well as between malignant and benign ovarian-derived cells (Desir et al., 2016). To further investigate the notion that there might be an equivalent Dunbar's number for TNTs that are formed *in vitro*, we reanalyzed the time-lapse imaging we had produced that documented TNT formation between cancer cells. As an example, we conducted a frame-by-frame examination of one of our time-lapse videos of malignant (Mg63.2-GFP-expressing osteosarcoma cell line) cells cocultured with osteoblast (the hFOB cell line stained with red DiI lipophilic dye) cells characteristic of the bone matrix (Figure 3, Supplementary Video 1). Each image represented a time frame of 30 min, over a total time of 40 h of cell culture. There was a range of average duration of TNTs—the time length of the intercellular “conversations” taking place—of 30 min to 2 h, but the vast majority of TNTs lasted no longer than 30 min (136/168 or >80%). The total percentage of TNTs that lasted 1–2 h was cumulatively small, at ~19% (32/168), and none lasted longer than 2 h. Interestingly, the overall proportion of cells that developed intercellular interactions with TNTs was small (7%) within the first 10 h in culture. However, between 10 h and 40 h, while there was a wide fluctuation between frames in some periods (range of TNTs = 0–8; range of ratio of cells with TNTs = 0–0.266), the average percentage of cells with TNTs tended to equilibrate despite this range of fluctuation throughout this period. The mean percentage of cells that formed TNTs was 3.5% within the first 10 h but 9.1% during the remainder of the 40-h period. In terms of the absolute numbers of TNTs, the mean was 1.05 TNTs within the first 10 h and 2.75 during the remainder of the 40-h period.

This preliminary analysis supports the notion that while TNTs are finite and dynamic structures, the overall ecosystem of semi-confluent cells in culture generally equilibrates to an overall stable number of TNTs at any given time. Whether this number is the “cellular equivalent” of a Dunbar's number is speculative, it underscores the role of systems biology as a key element in the development and survival of the overall tumor ecosystem. While it is likely that the actual number and percentages will vary from cell type to cell type, the concept that TNTs—as conduits of intercellular interactions and communication—may actually follow similar rate-limiting steps as social interactions is fascinating and further open the door to viewing the function and mechanisms that support TNTs in a new light.

## Ultrastructure of TNTs: are they single or multi-lane cellular highways or can they also act as intercellular bridge tracks for cargo surfing?

**Figure 4:**
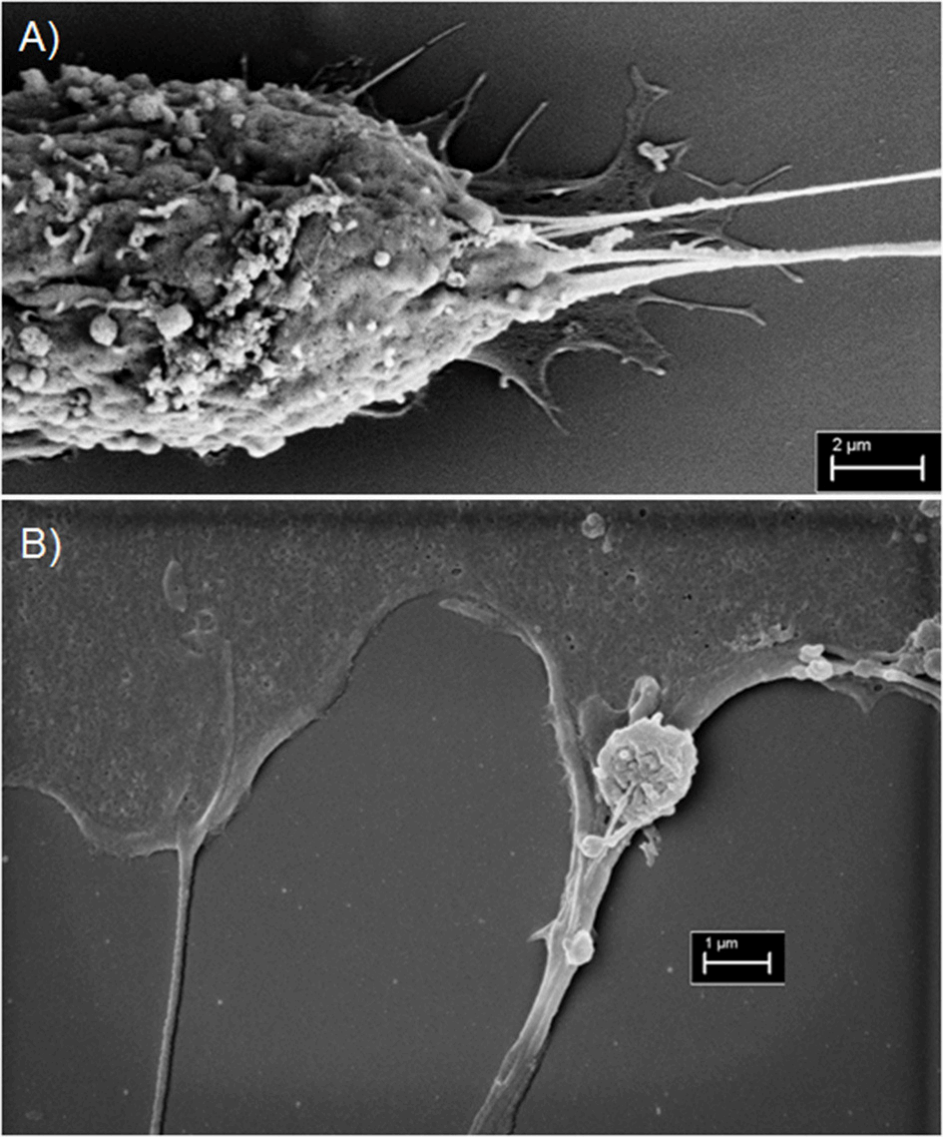
Scanning electron microscopy imaging of TNTs in MSTO-211H malignant mesothelioma cells. **(A)** Electron micrograph revealing multiple insertion points vs. points of extrusion of TNTs in the membrane of a MSTO cell (Scale bar = 2 μm; Magnification 14.62 k X; width = 20.52 μm). **(B)** A TNT is seen on the left, and on the right is a TNT-like protrusion vs. filopodia/invadopodial extension. An extracellular vesicle is overlying the extension on the right (Scale bar = 1 μm; Magnification 24.69 k X; width = 12.15 μm). Materials and Methods section for experiments shown in the figure is available in the Supplementary Material.

An ongoing debate that is central to the function, mechanism, and structural biology of TNTs is whether they are open-ended or closed structures. It is clear that TNTs are conduits that are capable of mediating cell-to-cell spread of cargo. Whether they are truly “tunneled” at both ends, remains a matter of debate. For that reason, in some studies the term “membrane nanotubes” or the equivalent is the preferred nomenclature. Advances in high-resolution microscopy are beginning to address these questions and will be crucial in the accurate evaluation of TNTs in the *in vivo* setting (Lou et al., 2017). In our initial studies, using malignant pleural mesothelioma as a model system, we reported from electron microscopic imaging that some TNTs had multiple insertion points in the cell membrane (Lou et al., 2012). By EM, we also identified single or multiple cable-like insertions that stem from the cell membrane (Figure 4A). Although we assumed that these short strands form the base of TNTs and merge into a single thicker TNT, we also considered that each of these strands represent independent TNTs that ultimately run parallel to each other on their way to connecting distant cells. Such a scenario might explain the heterogeneity of widths seen in TNTs across different cell types (across cancers and between cancer and non-cancer cells) as well as differences seen between nanotubes *in vitro* and tumor microtubes seen in *in vivo* tumor models (Osswald et al., 2015; Jung et al., 2017; Weil et al., 2017). A recent preprint article presented strong evidence supporting this concept using cryo-correlative light- and electron microscopy (CLEM) (Sartori-Rupp et al., 2018). The definition of what is and what is not a TNT continues to remain a bit of a controversy.

There is also precedence for the notion that if some nanotubes are indeed membranous but not necessarily tunneled, then cargo can still be transported by utilizing the tubes as “tracks” to guide the way to recipient cells. A prime example is retroviral virion particles “surfing” along the outer surface of TNT-like filopodial bridges as a means for intercellular transport (Sherer et al., 2007). Our work on TNTs in malignant mesothelioma showed that tumor cell-derived exosomes stimulated formation of more TNTs in this cell type; furthermore, using time-lapse fluorescence microscopy, we visualized exosomes tracking along and/or within these TNTs to connecting cells (Thayanithy et al., 2014a). The concept of exosomal/extracellular vesicles (ECVs) transferring within TNTs is not entirely new and is supported by work from other labs as well (Hood et al., 2009; Mineo et al., 2012). By scanning EM, we have captured at least one instance that further distinguished TNTs at their insertion/extrusion point in the membrane. What was most noticeable, however, was an adjoining TNT-like protrusion that appeared to be “carrying” an ECV (or microvesicle) that adhered to this protrusion by two short cable-like structures (Figure 4B). We speculated that this finding represents a form by which ECV cargo tracks along TNTs or similar cell extensions to more efficiently move from cell-to-cell. Overall, the refined use of EM and other high-resolution microscopic techniques will better elucidate the topography, landscape and interaction of cells, TNTs, and ECVs within the tumor ecosystem.

## Overexpression of CSPG4 is associated with increased rate of TNT formation

The search for structural or functional markers of TNTs remains a significant challenge. We have theorized, based on our experience so far, that while there are commonalities between TNTs in different cell types, there may also be unique structural features of TNTs that are upregulated in specific disease states, including cancer. One of the best characterized potential biomarkers of TNTs, to date, is M-Sec (TNF-aip2), which has been examined primarily in macrophages and other immune cells (Hase et al., 2009; Ohno et al., 2010; Schiller et al., 2013). We found that M-Sec was also upregulated in malignant mesothelioma cells cultured in conditions conducive to upregulation of TNTs (Ady, 2014). Thus, this marker may be the closest universal candidate that has been characterized, to date, across multiple cell types. Other standard components of cellular actin-based machinery, such as Cdc42 and Rac1, have also been associated with TNT formation (Hanna et al., 2017).

We have reported that mesothelioma cells that form TNTs are enriched in lipid rafts as compared with cells in coculture that do not form TNTs (Thayanithy et al., 2014a). Lipid rafts are cholesterol microdomains that aggregate at the intracytoplasmic domain in regions on the invasive leading edge of cells and have, therefore, been implicated in malignant invasion. We hypothesized that similar cell surface markers involved in cell migration and invasion might also be associated with and possibly stimulated TNT formation. Thus, we selected CSPG4, also known as neuron-glial antigen 2 or NG2. A transmembrane proteoglycan, CSPG4 plays a key role in stabilizing cell-substratum interactions in early melanoma cell invasion. Most importantly, it is overexpressed in mesothelioma but not in normal mesothelium (Rivera et al., 2012) and plays a role in mediating pericyte interaction with endothelial cells. Ablation of CSPG4/NG2 in a breast cancer animal model resulted in decreased progression and development of vasculature (Gibby et al., 2012). Targeting CSPG4 using monoclonal antibodies decreases mesothelioma cell invasiveness as well, and it has been proposed as a potential target for the treatment of mesothelioma, breast cancer, and melanoma (Wang et al., 2010; Price et al., 2011; Yu et al., 2011; Rivera et al., 2012). On the basis of our findings in mesothelioma, we concluded that the association of certain cellular markers such as lipid rafts with TNTs is an indicator of cell invasion (i.e., CSPG4) and that this leads to increased formation of TNTs.

**Figure 5:**
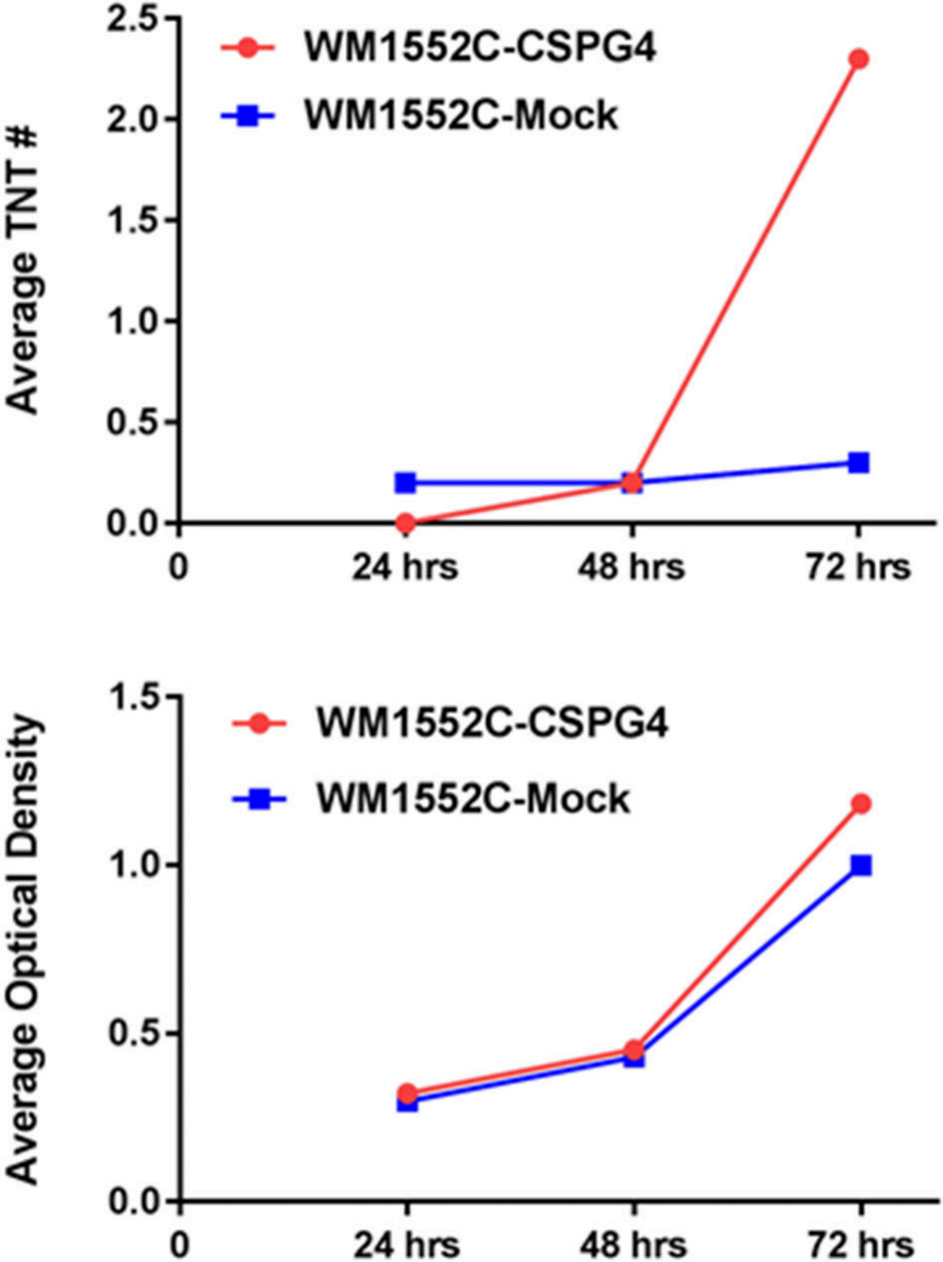
Melanoma cells that overexpress CSPG4 form TNTs at a higher rate over time. We quantified TNTs between WM1552C-CSPG4 and WM1552C-mock cells at 24 h, 48 h, and 72 h. The average number of TNTs per time point is shown **(Top)** as well as cell proliferation measured as average optical density **(Bottom)**. Materials and Methods section for experiments shown in the figure is available in the Supplementary Material.

To test this hypothesis, we used the melanocyte-derived radial growth phase melanoma cell line WM-1552, which was transfected to stably express CSPG4 (Yang et al., 2004) and was designated as WM1552-CSPG4. The expression of CSPG4 in WM1552 cells causes enhanced cell adhesion and spreading, increased motility, enhanced epithelial-to-mesenchymal transition (EMT), anchorage independent growth, and tumorigenic potential compared with the CSPG4 negative mock transfected counterparts (Yang et al., 2009; Price et al., 2011). In the current study, we compared TNT formation in WM1552c mock transfectants lacking CSPG4 with WM1552c CSPG4-expressing melanoma cell lines. We cultured WM1552 mock and CSPG4 cells separately in low-serum, hyperglycemic RPMI medium (2.5% FCS, 50 mM glucose) and counted TNTs manually at 24-h intervals for 96 h. To assess the rate of cellular proliferation, we also determined the relative absorbance of a surrogate measure of cell growth over time. We detected significantly higher numbers of TNTs at 96 h in WM1552-CSPG4 culture as compared with WM1552-mock cells (Figure 5). This increase in TNT formation was independent of two-dimensional cell growth. After accounting for a higher cellular absorbance rate for the former cell line as a measure of cell proliferation, TNT/absorbance remained significantly higher for WM1552-CSPG4 (1.2-fold higher or 21% higher, at 72 h), suggesting an association and potential stimulatory role of CSPG4 in the formation of TNTs. Functionally, CSPG4 is localized on the tips of filopodia and stimulates key oncogenic signaling pathways, including focal adhesion kinase, constitutively activated BRAF/Erk 1,2 and Rho family GTPases, all of which could be important for the stimulation of TNT formation (Price et al., 2011). Furthermore, the function of CSPG4 requires the cytoplasmic tail of the core protein. Thus, studies that interrogate CSPG4 may shed considerable insight into the molecular mechanisms that govern TNT formation (Price et al., 2011). Since CSPG4 is a potential target, studies that interrogate mechanisms of CSPG4 function may also positively identify the importance of TNTs as a clinical target in malignant progression.

## The question of active vs. passive diffusion: the myosin chaperone UNC-45A correlates with TNT formation for a limited time, but is inversely associated with TNT formation after 48 h in culture

There remain many questions regarding the active vs. passive nature of intercellular cargo transport that is mediated by TNTs. Discovery of a defining molecular component that is specific to TNTs remains elusive, and it is conceivable that a single one may not exist. The prevailing thought is that these filamentous actin-based cell protrusions employ the myosin motor complex in a fashion similar to other well-established modes of actin-based machinery.

**Figure 6:**
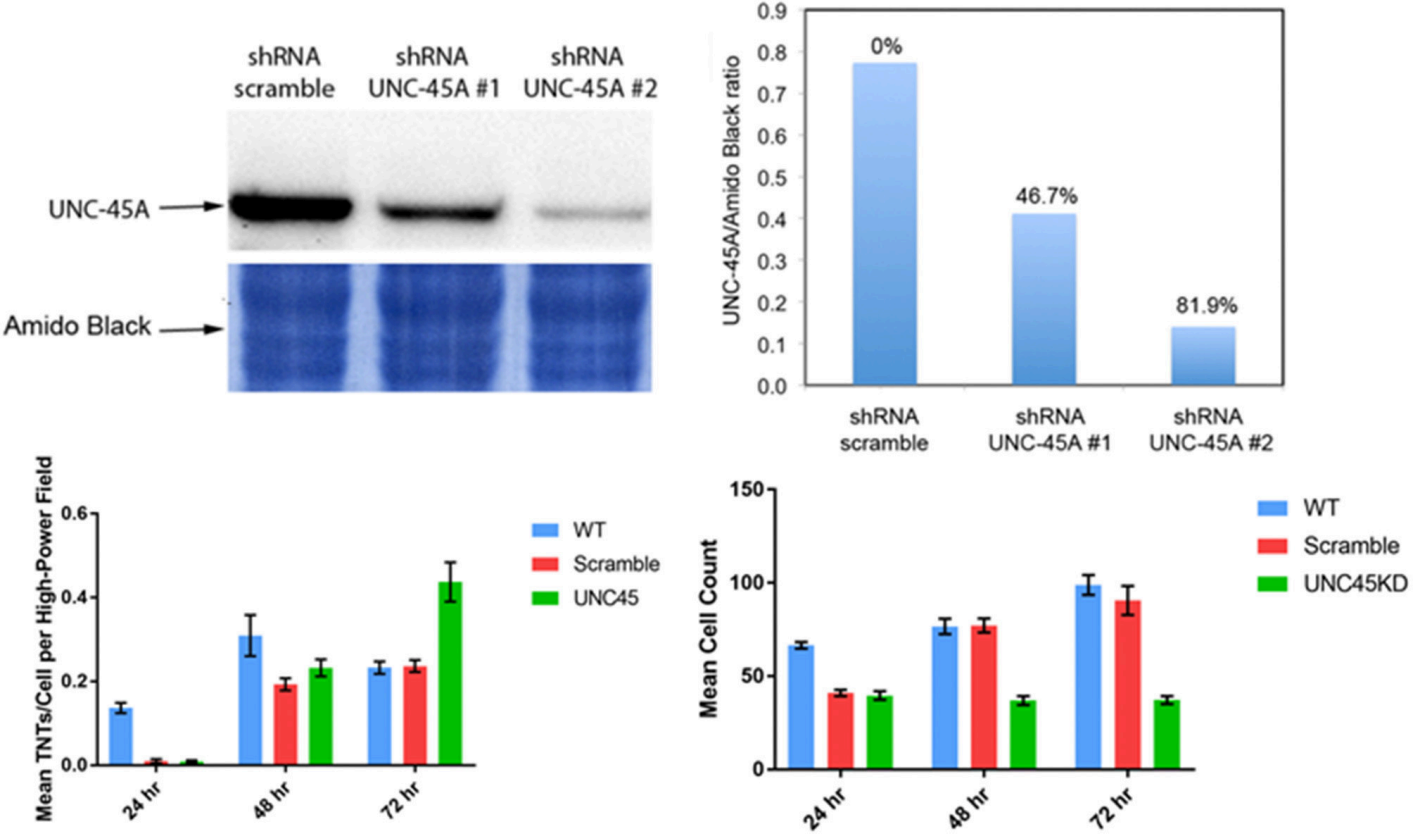
Knockdown of the UNC-45A myosin motor protein chaperone diminishes TNT formation, but this finding is limited in duration. Upper left: western blot demonstrating effective knockdown of UNC-45A using shRNA. Based on these results, shRNA #2 was used in our study. Upper right: quantitation of effective UNC45-A knockdown by shRNA #1 and #2. Lower left: mean number of TNTs at 24 h, 48 h, and 72 h in wild type SKOV3 ovarian cancer cells compared with cells transfected with the scramble and UNC-45A shRNA #2. Lower right: mean cell count for the same conditions and time points. Materials and Methods section for experiments shown in the figure is available in the Supplementary Material.

UNC-45A is a chaperone protein that has been well characterized for its function in the assembly and maintenance of the myosin II motor complex. It has been shown to be overexpressed in serous ovarian carcinomas and is associated with significantly increased cancer cell proliferation as well as motility (Bazzaro et al., 2007). Furthermore, the UNC-45A protein tracks at the leading edge of cells and is known to accumulate at the cell furrow during cytokinesis. We postulated that as TNTs arise at the cell edge, they might utilize UNC-45 for formation and maintenance. Thus, we examined TNT formation in ovarian cancer cells with intact UNC-45A expression as compared with the same cell line (SKOV3) with shRNA knockdown of UNC-45A; a scramble version of the lentivirus offered an additional form of control. We quantified the number of TNTs and cells in 20X fields of view to establish the TNT index (number of TNTs/cell over time), a method we have described in detail in other publications (Ady, 2014). Interestingly, we found that while the average number of TNTs per cell was significantly lower in the UNC-45A-knockdown group at 24 h and 48 h, by 72 h the reverse was seen, as on average there were more TNTs/cell among the cells in which ~80% of UNC-45A had been suppressed via shRNA knockdown (Figure 6). This finding was initially unexpected, but, upon further analysis, it made sense in the overall context based on the known role of UNC-45A in modulating cellular motility and proliferation.

This finding provides stimulus for examining the time course of TNTs and better understanding their niche, not just spatially but also in time. Tunneling nanotubes are most prolific in subconfluent cell cultures. As cultures become increasingly confluent, the physical space between cells is diminished, and there is less need for TNTs to form bridges between these cells. Knockdown of UNC-45A continued to suppress cell proliferation by 72 h; in comparison with scramble and wild type, the ratio of the number of TNTs/cell was naturally higher (Figure 6). This observation provided potential insight into the notion that UNC-45A and other mediators of myosin motors might be essential to the early stages of TNT formation among non-crowded cell populations; however, its importance might diminish over time in more confluent or nearly-confluent cell populations and in terms of maintenance, rather than formation, of TNTs.

## hENT1 expression is inversely correlated with TNTs in pancreatic cancer

The tumor microenvironment comprises a vast and complex set of players that mediate intracellular and intercellular discussion. Important players of intercellular communication include diffusible soluble hormone signals, exosomes and other ECVs, and connexin-based gap junctions for cells in immediate proximity. In certain cancers, there are other forms of transporters. One such example is hENT1. Human equilibrative nucleoside transporter 1 is highly expressed in malignant pancreatic tumors, has a life cycle of 14 h, and has been associated with improved prognosis in pancreatic cancer (Nivillac et al., 2011), although there is no apparent correlation of hENT1 expression to survival (Poplin et al., 2013).

**Figure 7:**
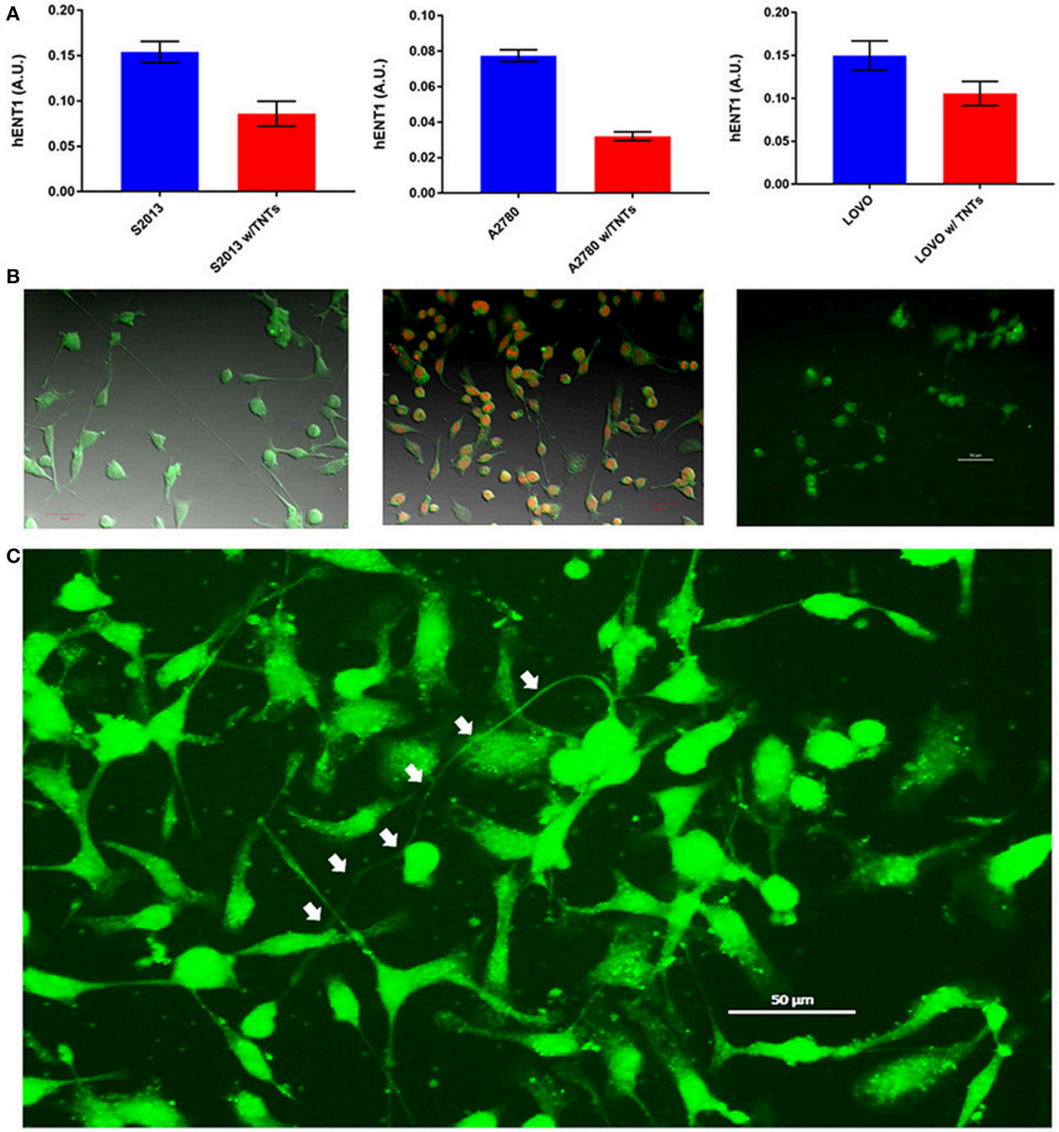
Cancer cells forming more TNTs demonstrate lower expression of hENT1. **(A)** hENT1 expression in S2013 pancreatic cancer cells, A270 ovarian cancer cells, and LOVO colon cancer cells with and without TNTs. **(B)** Corresponding images for each cell line are located beneath each graph. Cells were stained with immunofluorescent antibody that marks the expression of hENT1, and the expression was quantified as described. Scale bars = 50 μm for each of these panels. **(C)** The larger image on the bottom demonstrates a particularly long and curved TNT (indicated by white arrows) connecting hENT1-expressing S2013 cells. Materials and Methods section for experiments shown in the figure is available in the Supplementary Material.

The rationale for studying hENT is that it functions effectively as a transporter of nucleosides. More specifically, hENT1 permits intercellular transfer of chemotherapeutic agents such as the fluoropyrimidine gemcitabine, a standard of care drug that is potentially effective once it has successfully penetrated the dense stroma-rich microenvironment that is characteristic of this type of cancer. We recently reported that despite the especially dense nature of the pancreatic stromal reaction, TNTs could be detected in human pancreatic tumor tissue (Desir et al., 2018). We further postulated that if TNTs were the mediators of local and regional invasion and metastasis, then their presence would be inversely proportional to the expression of hENT1. As proof of concept, we examined a metastatic pancreatic carcinoma-derived cell line, S2013, for hENT1 expression using a fluorescent antibody. We detected focal expression of this transporter protein along the membrane, as expected. Quantifying its expression as arbitrary units (a.u.), and accounting for the area of each cell and the TNTs, we reported the expression in a fashion identical to our previously published study that examined lipid raft enrichment in TNT-forming cells (Thayanithy et al., 2014a). Consistent with our hypothesis, we found an inverse correlation between hENT1 expression and TNTs in these pancreatic cancer cells (Figure 7). In the S2013 cells, hENT1 expression was 1.8-fold higher in cells not forming TNTs than in cells in the same culture forming TNTs (*p* = 0.0023).

Although hENT1 is most often associated with pancreatic carcinomas, it is also present in other forms of cancer, including ovarian cancer (>90% expression) (Farré et al., 2004) and colon cancer (Liu et al., 2017). Thus, for comparison, we performed the same analysis in representative cell lines from these cancers as well. The results in those cell lines were consistent with our findings of inverse correlations of hENT1 to TNTs, indicating that TNTs may be indicative of more chemoresistant and/or more invasive cells. The expression of hENT1 was 2.4-fold higher in A2780 cells not forming TNTs as compared with those forming TNTs (*p* = 0.0126); this difference was 1.4-fold in LOVO cells, respectively (*p* = 0.0049).

It will be important not only to substantiate but also reconcile this finding with some of our own recent findings that TNTs are also conduits for mediating intercellular efflux of chemotherapeutic drugs (Desir et al., 2018). In fact, it has been reported that TNT-like tumor microtubes (TMs) seen in gliomas correlate with a higher degree of cellular invasion and drug resistance (Osswald et al., 2015, 2016; Jung et al., 2017; Weil et al., 2017).

## Localization of connexin proteins in relation to TNTs

Connexin channels that compose gap junctions mediate intercellular transfer of calcium and other small soluble factors; they are also not inherently separate from TNTs. Tunneling nanotubes and TMs have also been shown to mediate intercellular calcium flux effectively between malignant cells (Osswald et al., 2015; Lock et al., 2016). In fact, in both of these cited studies, the localization and attachment of connexins was found to be crucial to this TNT/TM-mediated process.

It is well established that gap junctions are downregulated in cancer cells undergoing EMT, a state that is strongly associated with stem cell-like properties and metastasis, which our own group showed was associated with significant upregulation in TNTs in malignant mesothelioma cells (Lou et al., 2012). Gap junctions have been reported to be localized at the tip of TNTs, for example in astrocytes (Wang and Gerdes, 2012), but reports, to date, have been inconsistent with this finding. The finding may be variable based on the timing of TNT formation, detection in its state at the time (e.g., in the midst of forming, transporting cargo, or disconnecting from recipient cells), the state of confluence of the cell culture being examined, and the cell type (malignant vs. non malignant or even heterogeneous between different cancer cell types). Furthermore, it is not yet firmly established whether gap junctions actually mediate the formation of TNTs from the plasma membrane and/or represent structural foundations of TNTs across all cell types. It remains to be determined whether connexins localize more at the base of TNTs where they emerge from the donor cell or at the point of entry into putative recipient cells.

**Figure 8:**
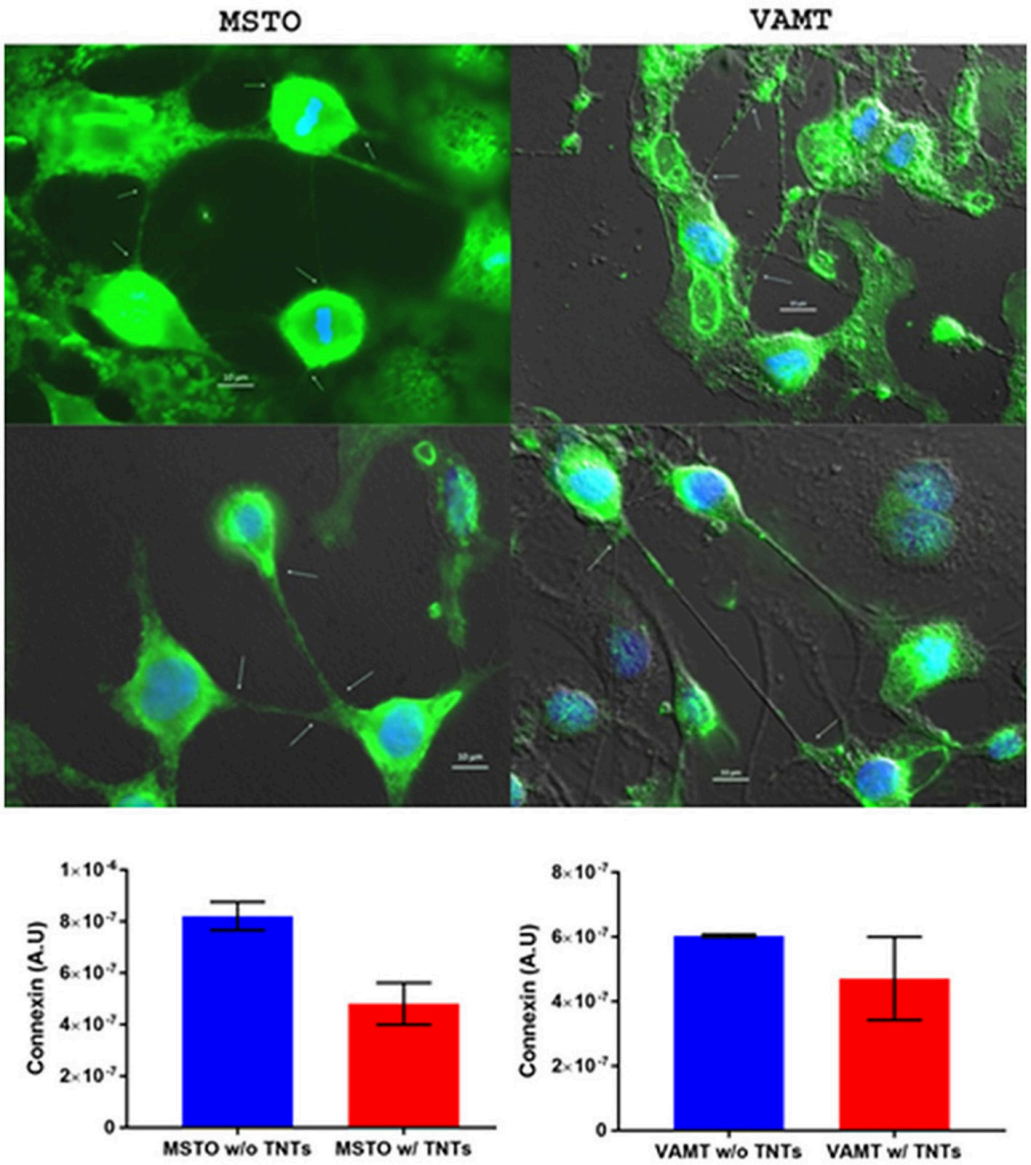
Connexin expression may be inversely correlated with TNT formation, and its expression co-localizes most prominently at the base and/or tips of TNTs. This experiment was performed using malignant mesothelioma cell lines MSTO-211H and VAMT. The upper panel shows representative images following fluorophore-tagged connexin staining of both cells lines. The lower panels show graphs of connexin expression in cells forming no TNTs (blue column) compared with those forming TNTs (red column). MSTO cell data are in the lower left graph, and VAMT data are in the lower right. Scale bars = 10 μm. Materials and Methods section for experiments shown in the figure is available in the Supplementary Material.

The question remains as to whether connexin expression differs quantitatively between cancer cells with or without TNTs. The answer is likely time-dependent; that is, dependent on the extent of cell confluency, distance between cells, and number of TNTs. We have extensively characterized TNT formation, function, and characterization in several cell lines of malignant mesothelioma (Lou et al., 2012; Ady, 2014, 2016; Thayanithy et al., 2014a). To examine connexin localization in these cells, we stained MSTO-211H and VAMT cells with immunofluorescent markers and performed inverted microscopy. A preliminary analysis was performed to quantify differences in connexin expression relative to expression in non-TNT forming cells. We found a 1.7-fold increase in connexin expression in MSTO cells not forming TNTs, as compared with cells forming TNTs; for the VAMT cell line, the difference was 1.28-fold (Figure 8). In the scope of systems biology, no component of the tumor matrix works independently. Rather, all of the components are dynamic and function as a unified process. For example, it is now established that exosomes interact synergistically with TNTs in cancer (Thayanithy et al., 2014a); connexin channels associated with TNTs may play a role in their initial formation and/or maintenance. It will be important to determine the variability between cancer cell types based on tissue of origin, state of EMT, and molecular status and also between cancer and non-cancer cell types.

## Conclusions

**Figure 9:**
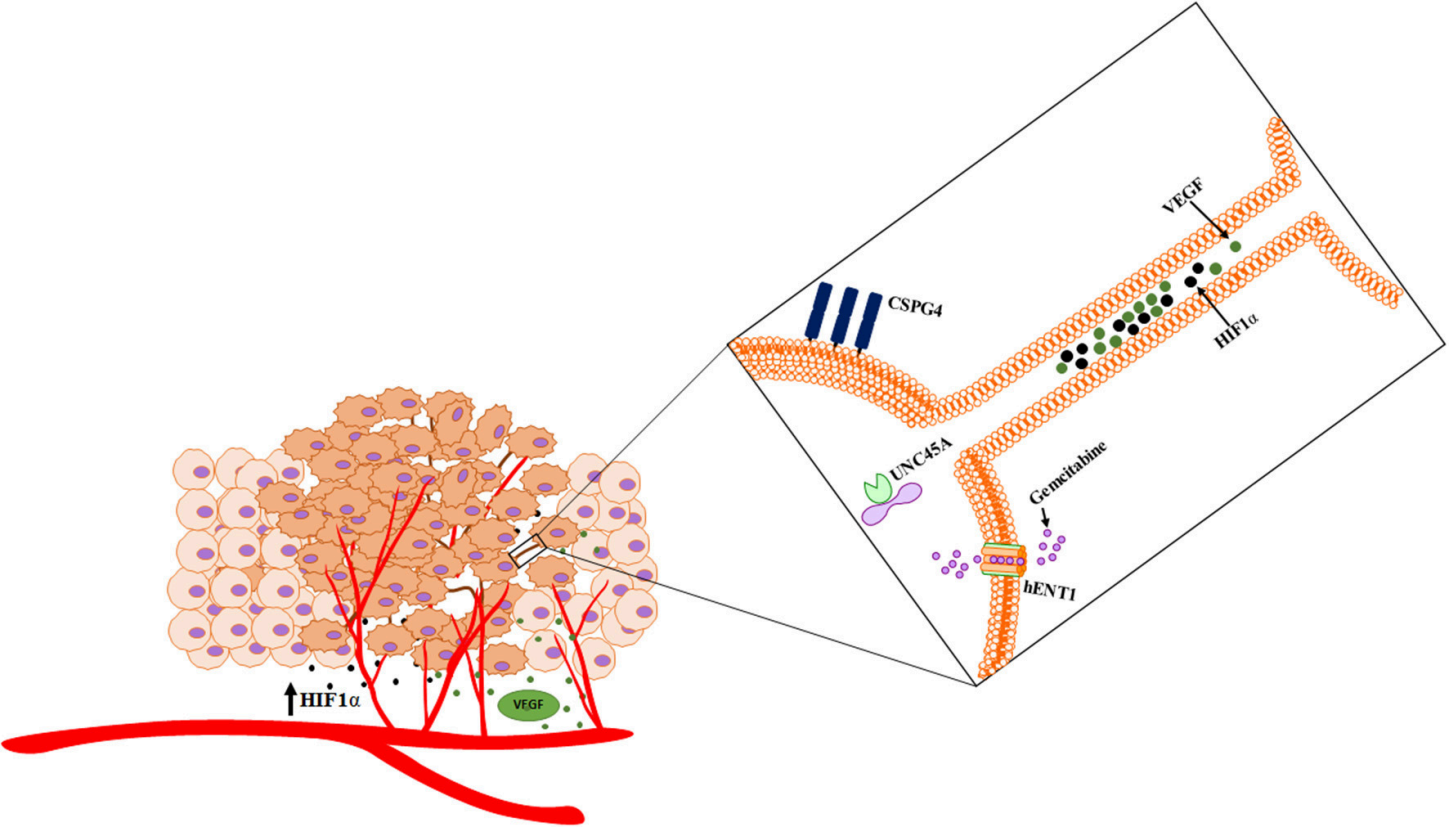
Schematic of cross talk via hypoxia-induced TNTs that facilitate the intercellular transfer of cargo. TNTs facilitate dynamic interplay that has implications in angiogenesis and other processes that are critical to the ecosystem.

Cancer cells cannot be studied in isolation, as they interact in a complex biological system. With the improved and rapidly expanded understanding of the function and importance of intercellular communication in modulating the tumor microenvironment, it is critical to investigate malignancy as a unique and continuously evolving ecosystem. These interactions can take many forms. Homotypic interactions of malignant cells are mediated via TNTs, exosomes, gap junctions, and a wide ranging list of diffusible and soluble factors. Heterotypic interactions include interactions of the extracellular matrix with malignant cells, vascular endothelium with cancer, immune cells with cancer, and hematologic components with cancer. Altogether, these events comprise a spectrum of intratumoral interactions that reprogram cells for invasion, metastasis, and emergence of resistance to treatment. The interplay and dynamics of the subtopics that we have begun to examine here are illustrated in the accompanying schematic (Figure 9). Perturbations to this complex ecosystem, such as those provided by drug- or radiation-induced injuries, can instead induce cellular stress responses that in turn increase the extent of any or all of these inflammatory interactions. Increased characterization of TNTs and their role in cancer cell invasion and chemoresistance over the past decade continues to provide novel mechanistic insights into how the heterogeneous tumor microenvironment adapts and evolves over time. Continued work in this exciting field, including concepts described here and elsewhere, will clarify to what extent TNTs play an important role in mediating the tumor ecosystem.

## Author contributions

EL wrote the initial drafts of the manuscript. EL, EZ, AS, SD, PW, YI, JM, and MB provided initial analysis of the data and prepared the figures. EL, SS, JM, MB, and CS performed further analysis and interpretation of the data and wrote subsequent drafts of the manuscripts. All authors reviewed and approved the final submitted manuscript.

### Conflict of interest statement

The authors declare that the research was conducted in the absence of any commercial or financial relationships that could be construed as a potential conflict of interest.

## Supplementary material

The Materials and Methods section for the experiments described in this paper is located in the Supplementary Material of this article, which can be found online at: https://www.frontiersin.org/articles/10.3389/fcell.2018.00095/full#supplementary-material

Supplementary Video 1 Time-lapse video of a coculture of Mg63.2 osteosarcoma cells with hFOB osteoblast cells.

## References

[B1] Ady J. (2016). Tunneling nanotubes: an alternate route for propagation of the bystander effect following oncolytic viral infection. Mol. Ther. Oncolyt. 3:16029. 10.1038/mto.2016.29 27933314 PMC5142513

[B2] Ady J. W. (2014). Intercellular communication in malignant pleural mesothelioma: properties of tunneling nanotubes. Front. Physiol. 5:400. 10.3389/fphys.2014.00400 25400582 PMC4215694

[B3] Al Heialy S. Zeroual M. Farahnak S. McGovern T. Risse P. A. Novali M.. (2015). Nanotubes connect CD4+ T cells to airway smooth muscle cells: novel mechanism of T cell survival. J. Immunol. 194, 5626–5634. 10.4049/jimmunol.1401718 25934863

[B4] Aravalli R. N. Cressman E. N. Steer C. J. (2012). Hepatic differentiation of porcine induced pluripotent stem cells *in vitro*. Vet. J. 194, 369–374. 10.1016/j.tvjl.2012.05.013 22749116

[B5] Banerjee S. Thayanithy V. Sangwan V. Mackenzie T. N. Saluja A. K. Subramanian S. (2013). Minnelide reduces tumor burden in preclinical models of osteosarcoma. Cancer Lett. 335, 412–420. 10.1016/j.canlet.2013.02.050 23499892 PMC4386634

[B6] Bazzaro M. Santillan A. Lin Z. Tang T. Lee M. K. Bristow R. E.. (2007). Myosin II co-chaperone general cell UNC-45 overexpression is associated with ovarian cancer, rapid proliferation, and motility. Am. J. Pathol. 171, 1640–1649. 10.2353/ajpath.2007.070325 17872978 PMC2043524

[B7] Chauveau A. Aucher A. Eissmann P. Vivier E. Davis D. M. (2010). Membrane nanotubes facilitate long-distance interactions between natural killer cells and target cells. Proc. Natl. Acad. Sci. U.S.A. 107, 5545–5550. 10.1073/pnas.0910074107 20212116 PMC2851811

[B8] Desir S. Dickson E. L. Vogel R. I. Thayanithy V. Wong P. Teoh D.. (2016). Tunneling nanotube formation is stimulated by hypoxia in ovarian cancer cells. Oncotarget. 7, 43150–43161. 10.18632/oncotarget.9504 27223082 PMC5190014

[B9] Desir S. O'Hare P. Vogel R. I. Sperduto W. Sarkari A. Dickson E.. (2018). Chemotherapy-Induced tunneling nanotubes mediate intercellular drug efflux in pancreatic cancer. Sci. Rep. 8:9484. 10.1038/s41598-018-27649-x 29930346 PMC6013499

[B10] Doganer B. A. Yan L. K. Q. Youk H. (2016). Autocrine signaling and quorum sensing: extreme ends of a common spectrum. Trends Cell Biol. 26, 262–271. 10.1016/j.tcb.2015.11.002 26671200

[B11] Dossey L. (2017). Is friendship limited? An inquiry into dunbars number. Explore 13, 1–5. 10.1016/j.explore.2016.10.008 27890520

[B12] Eugenin E. A. Gaskill P. J. Berman J. W. (2009). Tunneling nanotubes (TNT) are induced by HIV-infection of macrophages: a potential mechanism for intercellular HIV trafficking. Cell Immunol. 254, 142–148 10.1016/j.cellimm.2008.08.005 18835599 PMC2701345

[B13] Farré X. Guillén-Gómez E. Sánchez L. Hardisson D. Plaza Y. Lloberas J.. (2004). Expression of the nucleoside-derived drug transporters hCNT1, hENT1 and hENT2 in gynecologic tumors. Int. J. Cancer 112, 959–966. 10.1002/ijc.20524 15386342

[B14] Geddings J. E. Mackman N. (2013). Tumor-derived tissue factor-positive microparticles and venous thrombosis in cancer patients. Blood 122, 1873–1880. 10.1182/blood-2013-04-460139 23798713 PMC3772497

[B15] Gibby K. You W. K. Kadoya K. Helgadottir H. Young L. J. Ellies L. B.. (2012). Early vascular deficits are correlated with delayed mammary tumorigenesis in the MMTV-PyMT transgenic mouse following genetic ablation of the NG_2_ proteoglycan. Breast Cancer Res. 14:R67. 10.1186/bcr3174 22531600 PMC3446402

[B16] Gousset K. Marzo L. Commere P. H. Zurzolo C. (2013). Myo10 is a key regulator of TNT formation in neuronal cells. J. Cell Sci. 126(Pt 19), 4424–4435. 10.1242/jcs.129239 23886947

[B17] Gousset K. Schiff E. Langevin C. Marijanovic Z. Caputo A. Browman D.. (2009). Prions hijack tunnelling nanotubes for intercellular spread. Nat. Cell Biol. 11, 328–336. 10.1038/ncb1841 19198598

[B18] Hanna S. J. McCoy-Simandle K. Miskolci V. Guo P. Cammer M. Hodgson L.. (2017). The Role of Rho-GTPases and actin polymerization during macrophage tunneling nanotube biogenesis. Sci. Rep:7:8547. 10.1038/s41598-017-08950-7 28819224 PMC5561213

[B19] Hase K. Kimura S. Takatsu H. Ohmae M. Kawano S. Kitamura H.. (2009). M-Sec promotes membrane nanotube formation by interacting with Ral and the exocyst complex. Nat. Cell Biol. 11, 1427–1432. 10.1038/ncb1990 19935652

[B20] Hood J. L. Pan H. Lanza G. M. Wickline S. A. (2009). Paracrine induction of endothelium by tumor exosomes. Lab Invest. 89, 1317–1328. 10.1038/labinvest.2009.94 19786948 PMC3316485

[B21] Iglic A. Lokar M. Babnik B. Slivnik T. Veranic P. Hägerstrand H.. (2007). Possible role of flexible red blood cell membrane nanodomains in the growth and stability of membrane nanotubes. Blood Cells Mol. Dis. 39, 14–23. 10.1016/j.bcmd.2007.02.013 17475520

[B22] Iizuka Y. Cichocki F. Sieben A. Sforza F. Karim R. Coughlin K.. (2015). UNC-45A is a nonmuscle myosin IIA chaperone required for NK cell cytotoxicity via control of lytic granule secretion. J. Immunol. 195, 4760–4770. 10.4049/jimmunol.1500979 26438524 PMC5189640

[B23] Iizuka Y. Mooneyham A. Sieben A. Chen K. Maile M. Hellweg R.. (2017). UNC-45A is required for neurite extension via controlling NMII activation. Mol. Biol. Cell 28, 1337–1346. 10.1091/mbc.e16-06-0381 28356421 PMC5426848

[B24] Jung E. Osswald M. Blaes J. Wiestler B. Sahm F. Schmenger T. (2017). Tweety-homologue 1 drives brain colonization of gliomas. J. Neurosci. 37, 6837–6850. 10.1523/JNEUROSCI.3532-16.2017 28607172 PMC6705725

[B25] Junt T. Schulze H. Chen Z. Massberg S. Goerge T. Krueger A.. (2007). Dynamic visualization of thrombopoiesis within bone marrow. Science 317, 1767–1770. 10.1126/science.1146304 17885137

[B26] Lachambre S. Chopard C. Beaumelle B. (2014). Preliminary characterisation of nanotubes connecting T-cells and their use by HIV-1. Biol. Cell 106, 394–404. 10.1111/boc.201400037 25130443

[B27] Liu Y. Zuo T. Zhu X. Ahuja N. Fu T. (2017). Differential expression of hENT1 and hENT2 in colon cancer cell lines. Genet. Mol. Res. 16:gmr16019549. 10.4238/gmr16019549 28218790

[B28] Lock J. T. Parker I. Smith I. F. (2016). Communication of Ca(2+) signals via tunneling membrane nanotubes is mediated by transmission of inositol trisphosphate through gap junctions. Cell Calc. 60, 266–272. 10.1016/j.ceca.2016.06.004 27388952 PMC5035603

[B29] Lou E. Fujisawa S. Morozov A. Barlas A. Romin Y. Dogan Y.. (2012). Tunneling nanotubes provide a unique conduit for intercellular transfer of cellular contents in human malignant pleural mesothelioma. PLoS ONE 7:e33093. 10.1371/journal.pone.0033093 22427958 PMC3302868

[B30] Lou E. Gholami S. Romin Y. Thayanithy V. Fujisawa S. Desir S.. (2017). Imaging tunneling membrane tubes elucidates cell communication in tumors. Trends Cancer 3, 678–685. 10.1016/j.trecan.2017.08.001 28958386

[B31] Mineo M. Garfield S. H. Taverna S. Flugy A. De Leo G. Alessandro R.. (2012). Exosomes released by K562 chronic myeloid leukemia cells promote angiogenesis in a Src-dependent fashion. Angiogenesis 15, 33–45. 10.1007/s10456-011-9241-1 22203239 PMC3595015

[B32] Nivillac N. M. Bacani J. Coe I. R. (2011). The life cycle of human equilibrative nucleoside transporter 1: from ER export to degradation. Exp. Cell Res. 317, 1567–1579. 10.1016/j.yexcr.2011.03.008 21402067

[B33] Ohno H. Hase K. Kimura S. (2010). M-Sec: emerging secrets of tunneling nanotube formation. Commun. Integr. Biol. 3, 231–233. 10.4161/cib.3.3.11242 20714400 PMC2918763

[B34] Olumuyiwa-Akeredolu O. O. Pretorius E. (2015). Platelet and red blood cell interactions and their role in rheumatoid arthritis. Rheumatol. Int. 35, 1955–1964. 10.1007/s00296-015-3300-7 26059943

[B35] Onfelt B. Purbhoo M. A. Nedvetzki S. Sowinski S. Davis D. M. (2005). Long-distance calls between cells connected by tunneling nanotubules. Sci. STKE 2005:pe55. 10.1126/stke.3132005pe55 16333019

[B36] Osswald M. Jung E. Sahm F. Solecki G. Venkataramani V. Blaes J.. (2015). Brain tumour cells interconnect to a functional and resistant network. Nature 528, 93–98. 10.1038/nature16071 26536111

[B37] Osswald M. Solecki G. Wick W. Winkler F. (2016). A malignant cellular network in gliomas: potential clinical implications. Neuro. Oncol. 18, 479–485. 10.1093/neuonc/now014 26995789 PMC4799690

[B38] Pagès F. Mlecnik B. Marliot F. Bindea G. Ou F. S. Bifulco J.. (2018). International validation of the consensus Immunoscore for the classification of colon cancer: a prognostic and accuracy study. Lancet 391, 2128–2139. 10.1016/S0140-6736(18)30789-X 29754777

[B39] Park C. W. Zeng Y. Zhang X. Subramanian S. Steer C. J. (2010). Mature microRNAs identified in highly purified nuclei from HCT116 colon cancer cells. RNA Biol. 7, 606–614. 10.4161/rna.7.5.13215 20864815 PMC3073257

[B40] Pasquier J. Guerrouahen B. S. Al Thawadi H. Ghiabi P. Maleki M. Abu-Kaoud N.. (2013). Preferential transfer of mitochondria from endothelial to cancer cells through tunneling nanotubes modulates chemoresistance. J. Transl. Med. 11:94. 10.1186/1479-5876-11-94 23574623 PMC3668949

[B41] Pedicini L. Miteva K. T. Hawley V. Gaunt H. J. Appleby H. L. Cubbon R. M.. (2018). Homotypic endothelial nanotubes induced by wheat germ agglutinin and thrombin. Sci Rep. 8:7569. 10.1038/s41598-018-25853-3 29765077 PMC5953990

[B42] Plotnikov E. Y. Khryapenkova T. G. Galkina S. I. Sukhikh G. T. Zorov D. B. (2010). Cytoplasm and organelle transfer between mesenchymal multipotent stromal cells and renal tubular cells in co-culture. Exp. Cell Res. 316, 2447–2455. 10.1016/j.yexcr.2010.06.009 20599955

[B43] Poplin E. Wasan H. Rolfe L. Raponi M. Ikdahl T. Bondarenko I.. (2013). Randomized, multicenter, phase II study of CO-101 versus gemcitabine in patients with metastatic pancreatic ductal adenocarcinoma: including a prospective evaluation of the role of hENT1 in gemcitabine or CO-101 sensitivity. J. Clin. Oncol. 31, 4453–4461. 10.1200/JCO.2013.51.0826 24220555

[B44] Poulter N. S. Pollitt A. Y. Davies A. Malinova D. Nash G. B. Hannon M. J.. (2015). Platelet actin nodules are podosome-like structures dependent on Wiskott-Aldrich syndrome protein and ARP2/3 complex. Nat. Commun. 6:7254. 10.1038/ncomms8254 26028144 PMC4458878

[B45] Price M. A. Colvin Wanshura L. E. Yang J. Carlson J. Xiang B. Li G.. (2011). CSPG4, a potential therapeutic target, facilitates malignant progression of melanoma. Pigment Cell Melanoma Res. 24, 1148–1157. 10.1111/j.1755-148X.2011.00929.x 22004131 PMC3426219

[B46] Rivera Z. Ferrone S. Wang X. Jube S. Yang H. Pass H.. (2012). CSPG4 as a target of antibody-based immunotherapy for malignant mesothelioma. Clin. Cancer Res. 18, 5352–5363. 10.1158/1078-0432.CCR-12-0628 22893632 PMC3463742

[B47] Rustom A. Saffrich R. Markovic I. Walther P. Gerdes H. H. (2004). Nanotubular highways for intercellular organelle transport. Science 303, 1007–1010. 10.1126/science.1093133 14963329

[B48] Sartori-Rupp A. Cordero Cervantes D. Pepe A. Delage E. Gousset K. Corroyer-Dulmont S. C. (2018). Mapping of TNTs using correlative cryo-electron microscopy reveals a novel structure. bioRxiv. [preprint] 10.1101/342469 PMC6341166 30664666

[B49] Sarver A. L. Li L. Subramanian S. (2010). MicroRNA miR-183 functions as an oncogene by targeting the transcription factor EGR1 and promoting tumor cell migration. Cancer Res. 70, 9570–9580. 10.1158/0008-5472.CAN-10-2074 21118966

[B50] Schertzer J. W. Whiteley M. (2011). Microbial communication superhighways. Cell 144, 469–470. 10.1016/j.cell.2011.02.001 21335231 PMC3984534

[B51] Schiller C. Diakopoulos K. N. Rohwedder I. Kremmer E. von Toerne C. Ueffing M.. (2013). LST1 promotes the assembly of a molecular machinery responsible for tunneling nanotube formation. J. Cell Sci. 126(Pt 3), 767–777. 10.1242/jcs.114033 23239025

[B52] Schwertz H. Köster S. Kahr W. H. Michetti N. Kraemer B. F. Weitz D. A.. (2010). Anucleate platelets generate progeny. Blood 115, 3801–3809. 10.1182/blood-2009-08-239558 20086251 PMC2865870

[B53] Sherer N. M. Lehmann M. J. Jimenez-Soto L. F. Horensavitz C. Pypaert M. Mothes W. (2007). Retroviruses can establish filopodial bridges for efficient cell-to-cell transmission. Nat. Cell Biol. 9, 310–315. 10.1038/ncb1544 17293854 PMC2628976

[B54] Stone R. L. Nick A. M. McNeish I. A. Balkwill F. Han H. D. Sood A. K.. (2012). Paraneoplastic thrombocytosis in ovarian cancer. N. Engl. J. Med. 366, 610–618. 10.1056/NEJMoa1110352 22335738 PMC3296780

[B55] Swanepoel A. C. Pretorius E. (2013). Red blood cell and platelet interactions in healthy females during early and late pregnancy, as well as postpartum. Blood 121:3788. 10.1182/blood-2013-01-480053 23802272 PMC3650701

[B56] Thayanithy V. Babatunde V. Dickson E. L. Wong P. Oh S. Ke X.. (2014a). Tumor exosomes induce tunneling nanotubes in lipid raft-enriched regions of human mesothelioma cells. Exp. Cell Res. 323, 178–188. 10.1016/j.yexcr.2014.01.014 24468420 PMC4159162

[B57] Thayanithy V. Dickson E. L. Steer C. Subramanian S. Lou E. (2014b). Tumor-stromal cross talk: direct cell-to-cell transfer of oncogenic microRNAs via tunneling nanotubes. Transl. Res. 164, 359–365. 10.1016/j.trsl.2014.05.011 24929208 PMC4242806

[B58] Thayanithy V. O'Hare P. Wong P. Zhao X. Steer C. J. Subramanian S.. (2017). A transwell assay that excludes exosomes for assessment of tunneling nanotube-mediated intercellular communication. Cell Commun. Signal 15:46. 10.1186/s12964-017-0201-2 29132390 PMC5683209

[B59] van Rooy M. J. Pretorius E. (2016). Platelet interaction with erythrocytes and propensity to aggregation in essential thrombocythaemia. Lancet 387:1210. 10.1016/S0140-6736(14)62293-5 25982040

[B60] Wang X. Gerdes H. H. (2012). Long-distance electrical coupling via tunneling nanotubes. Biochim. Biophys. Acta 1818, 2082–2086. 10.1016/j.bbamem.2011.09.002 21930113

[B61] Wang X. Osada T. Wang Y. Yu L. Sakakura K. Katayama A.. (2010). CSPG4 protein as a new target for the antibody-based immunotherapy of triple-negative breast cancer. J. Natl. Cancer Inst. 102, 1496–1512. 10.1093/jnci/djq343 20852124 PMC2950168

[B62] Weil S. Osswald M. Solecki G. Grosch J. Jung E. Lemke D.. (2017). Tumor microtubes convey resistance to surgical lesions and chemotherapy in gliomas. Neuro. Oncol. 19, 1316–1326. 10.1093/neuonc/nox070 28419303 PMC5596180

[B63] Yang J. Price M. A. Li G. Y. Bar-Eli M. Salgia R. Jagedeeswaran R.. (2009). Melanoma proteoglycan modifies gene expression to stimulate tumor cell motility, growth, and epithelial-to-mesenchymal transition. Cancer Res. 69, 7538–7547. 10.1158/0008-5472.CAN-08-4626 19738072 PMC2762355

[B64] Yang J. Price M. A. Neudauer C. L. Wilson C. Ferrone S. Xia H.. (2004). Melanoma chondroitin sulfate proteoglycan enhances FAK and ERK activation by distinct mechanisms. J. Cell. Biol. 165, 881–891. 10.1083/jcb.200403174 15210734 PMC2172406

[B65] Yu L. Favoino E. Wang Y. Ma Y. Deng X. Wang X. (2011). The CSPG4-specific monoclonal antibody enhances and prolongs the effects of the BRAF inhibitor in melanoma cells. Immunol. Res. 50, 294–302. 10.1007/s12026-011-8232-z 21717063

[B66] Zhang L. Zhang Y. (2015). Tunneling nanotubes between rat primary astrocytes and C6 glioma cells alter proliferation potential of glioma cells. Neurosci. Bull. 31, 371–378. 10.1007/s12264-014-1522-4 25913038 PMC5563692

[B67] Zhang Y. Sime W. Juhas M. Sjölander A. (2013). Crosstalk between colon cancer cells and macrophages via inflammatory mediators and CD47 promotes tumour cell migration. Eur. J. Cancer 49, 3320–3334. 10.1016/j.ejca.2013.06.005 23810249

