# Supplementary material for: Cellular and Molecular Networking Within the Ecosystem of Cancer Cell Communication via Tunneling Nanotubes
Source: Front Cell Dev Biol. 2018 Oct 2;6:95. doi: 10.3389/fcell.2018.00095 (PMC6176212; doi:10.3389/fcell.2018.00095)
Supplement: Supplementary file 2 [file Table_1.docx]

**SUPPLEMENTARY INFORMATION**

**Materials and Methods**

*Cell lines and Materials*

Colon cancer cell lines SW480, HCT-116, and DLD-1, NIH 3T3 fibroblasts, adenoma cell line AAC1, the ovarian cancer cell line SKOV3, GFP-expressing human metastatic osteosarcoma cell line Mg63.2, and human osteoblast cell line hFOB were cultured and used as described previously (11, 60-64). The melanoma cell lines WM-1552 mock (49) and WM-1552 CSPG4 were kindly provided by Dr. James McCarthy, and cultured in medium per established protocols. Malignant pleural mesothelioma cell lines MSTO-211H and VAMT were cultured and prepared for staining and assessment of TNTs as we have described previously (5, 25). Cell lines were authenticated using sequence tandem repeat genotype profiling (Johns Hopkins University, STR Profiling for Human Cell Line Authentication) and were also confirmed to be free of mycoplasma infection.

### **Culture conditions for TNT formation in normoxia and hypoxia**

Cells were cultured in a low-serum, high-glucose environment to determine the growth of TNTs as previously described (5, 25, 65). Briefly, the culture conditions consisted of RPMI-1640 medium with 2.5% FBS, 50 mM glucose, 1% P-S, 2% L-Glutamine, 10 nM ammonium lactate, and pH 6.6. For hypoxic conditions, cells were then placed in 10-cm cell culture plates and placed in a chamber containing 2% oxygen, 5% carbon dioxide, and 93% nitrogen.

### **Quantification of TNTs**

TNTs were identified as previously described (5, 25, 65). Briefly, these parameters included (i) lack of adherence to the substratum of tissue culture plates, including visualization of TNTs passing over adherent cells; (ii) TNTs connecting two cells or extending from one cell were counted if the width of the extension was estimated to be < 1000 nm; and (iii) a narrow base at the site of extrusion from the plasma membrane. Cellular extensions not clearly consistent with the above parameters were excluded. An Olympus IX70 inverted microscope (Olympus Corporation) with 20× objective lens was used to count TNTs and cells in 10 randomly chosen fields in each well.

**Cell proliferation assays**

Cell proliferation assays were performed using the Cell Counting Kit-8 (CCK8) per the manufacturer’s instructions. Cells were plated at a density of 3,300 cells per well in a 96-well plate in triplicate with 200 μl of medium in each well. Cells were allowed to grow in standard conditions. 10 μl of CCK8 reagent was placed in each well at 4 time points (24, 48, 72, and 96 hours) and left for incubation. Samples were then read in triplicate using a plate reader, and results were graphed using Prism.

**Time-lapse imaging**

DiI is a commercially available lipophilic dye that fluoresces in the red channels (Life Technologies, Carlsbad, CA). Cultures were prepared by co-culturing Mg63.2 human osteosarcoma cells expressing GFP in a 1:1 ratio with human osteoblasts from the hFOB cell line stained with DiI. Time-lapse imaging was performed using the Incucyte ZOOM system for live-cell imaging and analysis (Essen BioScience, Ann Arbor, Michigan). Environmental chambers were maintained at 37°C and 5% CO_2_. For the video provided as Supplementary Movie 1, brightfield images were taken every 30 minutes for 40 hours. Differential interference contrast and fluorescent imaging were performed concurrently.

*Imaging of HIF-1α and VEGF in TNTs*

Transfection of SKOV3 cells

Human Hypoxia Inducible Factor-1 Alpha subunit (HIF-1α) cDNA cloned under CMV promoter with a C-terminal green fluorescent protein (GFP) fusion (RGpCMV6-AC-GFP) was purchased from a commercial vendor, and plasmid was amplified after transformation into host bacteria following instructions of the manufacturer (Origene, Rockville, MD). The plasmid was transfected into SKOV3 cells using lipofectamine 2000 following standard protocols of the manufacturer (Life technologies, Carlsbad, CA). After 36 hours of transfection, transfected cells were monitored for GFP expression and harvested by standard trypsinization.

Co-culture and Live imaging of HIF1α/GFP-expressing SKOV3 cells with IOSE cells

IOSE (benign ovarian epithelial) cells were stained with lipophilic dye DiI and cultured with HIF-1α-transfected SKOV3 cells at a 1:1 ratio (10 x 10^5^ cells each) and plated in 35 mm petri dishes with glass bottom (MatTek corporation, Ashland, MA. Catalog no: P35G-1.5-20-C). Cells were allowed to adhere at standard cell culture conditions for 12 hours before imaging. Co-cultured SKOV3 and IOSE cells were imaged by live cell microscopy (VivaView, Olympus Corporation) at 20x objective every 10 minutes for 48 hours at phase and green and red fluorescence channels and analyzed using Metamorph software.

*Electron-Microscopic Imaging of Nanotubes*

To perform scanning EM, 1-3 x 10^6^ MSTO-211H cells were cultured on Thermanox plastic tissue culture 25 mm cover slips (Lux Scientific Corporation). The fixative — 2.5% glutaraldehyde/2% paraformaldehyde in 0.075 M sodium cacodylate buffer (pH 7.5; 10 ml, Electron Microscopy Sciences, Hatfield, PA) — was added directly to the overlying medium. Images were taken using a Zeiss Supra 25 Field Emission Scanning Electron Microscope. Please see the corresponding figure legend for details on image magnification and width for individual images.

*CSPG4-expressing Melanoma Cells*

To assess differences in TNTs between melanoma cells in which CSPG4 was expressed (WM1552-CSPG4) or knocked down (WM1552-Mock), we seeded 1x10^5^ cells per well in 6-well plates to quantify TNT formation at 24, 48, 72, and 96 hours. In parallel, the same numbers of cells were seeded in wells of 96-well plates to quantify absorbance as a surrogate for assessment of cell proliferation and metabolism as described in the “Cell proliferation assays” methods subsection.

*UNC-45A assessment and analysis*

SKOV3 ovarian carcinoma cells were cultured in DMEM supplemented with 10% fetal bovine serum. For UNC-45A silencing, scramble and UNC-45A shRNAs lentiviral supernatants were prepared and used to infect SKOV3 cells as we have previously described (66, 67). Differences in protein expression compared to native SKOV3 cells were confirmed using a standard Western blot. Amido black staining was performed to verify equal protein loading. Differences in average number of TNTs per cell were assessed as described above.

*hENT1 and connexin channel staining and analysis*

S2013 pancreatic carcinoma, LOVO colon cancer, and SKOV3 ovarian carcinoma cells were used for assessment of hENT, and MSTO-211H and VAMT malignant pleural mesothelioma cells were used for assessment of connexins. For both sets of experiments, cells were grown in chamber slides (Lab-Tek 155379), rinsed in PBS, and then fixed for 15 minutes in 4% paraformaldehyde, and washed 3 x 5 minutes with PBS (All subsequent washes are 3x5min in PBS). Cells were then permeabilized with 0.2% Tween/PBS for 10 minutes and washed again. Antigen retrieval was performed using 2N HCL for 10 min and then washed. They were then blocked with 3% Donkey Serum/PBST for 30 minutes and then washed.

For hENT1 staining, samples were then incubated overnight at 4ºC with hENT1 Ab (Rabbit Anti-Human hENT1 Monoclonal Ab (Clone SP120) Spring Bioscience cat# M4200)) at 1:50 dilution. For connexin staining, samples were incubated overnight at 4ºC with Connexin Ab (Santa Cruz Biotechnology, Inc.; Connexin 43 (CXN-6): sc-59949) at 1:200 dilution. For both protocols, the next day slides were washed, then incubated for 1 hour with secondary Ab (Alexa Fluor 488F(ab’)2 fragment of goat anti-rabbit IgG(H+L) at 1:500 from Molecular Probes cat#: A11070 [2mg/ml] stock), with 1% Donkey Serum in PBS. Samples were then washed and counterstained with Propidium Iodide [2ug/ml] for 5 minutes and then washed again. For connexin staining, the samples were then washed and counterstained with Hoechst Stain (BioRad, Hoechst 3334, Cat No 1351304) at 1:4000 for 5 minutes and then washed again. Coverslips were mounted using Invitrogen Prolong Gold antifade reagent and allowed to dry/cure overnight.

hENT1 images were taken at 20x using Zeiss Axio Observer Inverted Microscope, and connexin images were taken at 63x objective. Fluorescence was quantitated using Image J as previously described (25).
